# Adoption and use of the 7-1-7 metrics for effective detection, notification, and early response actions to public health events: A mixed-methods study in Cambodia, January 2023 to July 2025

**DOI:** 10.12688/wellcomeopenres.25041.2

**Published:** 2026-01-19

**Authors:** Sokly Mom, Sopheavy Seng, Hay Puthik Long, Kannamkottapilly Chandrasekharan Prajitha, Chanrasmey Pech, Sovann Sao, Sengdeourn Yi, Sovann Ly

**Affiliations:** 1 Department of Communicable Disease Control, Ministry of Health, Phnom Penh, 120101, Cambodia; 2 Cambodia Field Epidemiology Training Program, Ministry of Health, Phnom Penh, 120101, Cambodia; 3 International Union Against TB and Lung Disease, Paris, 75001, France; 4 Independent researcher, Phnom Penh, 12411, Cambodia

**Keywords:** 7-1-7 timeliness metrics, adoption, bottleneck, enabler, implementation research, public health events, outbreaks and response, surveillance

## Abstract

**Background:**

The 7-1-7 metrics with ≤7days for detection, ≤1 day for notification, ≤7 days to complete early response actions during public health outbreaks, enable early detection, notification, and rapid response. We evaluated the adoption, use of 7-1-7 metrics, and the bottlenecks and enablers for achieving the metrics in the public health sector of Cambodia.

**Methods:**

A sequential explanatory mixed-methods design was used. A cross-sectional study using a structured checklist was employed to assess the level of adoption (26 participants) and a cohort design to assess use of 7-1-7 metrics for the public health events reported between Jan’23 and Jul’2025. A qualitative descriptive study using 6 focus group discussions and 22 key informant interviews was conducted to explore the bottlenecks and enablers in 7-1-7 adoption and implementation.

**Results:**

Adoption of 7-1-7 metrics was achieved at national level and is in progress at the subnational level. Health system readiness, intersectoral coordination, capacity building, budget and resources, and digital data collection were major themes that influence adoption. Among 58 events, 26 were respiratory illnesses. The median(range) time was 6(<1-149)days for detection, with 36(62%) events meeting the target; less than 24 hours(0-4) for notification with 51(88%) events meeting the target; 1(1-101)day for early response with 50(86%) events meeting the target. Overall, 28(48%) events met all targets. Among 168 bottlenecks identified, 86(62%) were for detection, and among the 226 enablers identified, 94(42%) were related to early responses. Key bottlenecks included delayed care-seeking, low awareness among private health workers, and lack of community knowledge. Significant enablers included strong reporting lines and rapid coordinated response mechanisms.

**Conclusion:**

Cambodia has made substantial progress in adopting and implementing the 7-1-7 metrics. Delays in detection remain a key challenge; addressing this through enhanced risk communication, stronger private sector engagement, and improved surveillance capacity will strengthen Cambodia's overall outbreak preparedness.

## Plain language summary

Early detection, prompt notification, and timely response are essential for effective outbreak prevention and control. In partnership with the United States Centers for Disease Control and Prevention and Resolve to Save Lives, Cambodia’s Ministry of Health introduced the 7-1-7 timeliness tool in early 2023. This mixed-method study, conducted from 2023 to July 2025, evaluated the adoption and use of the 7-1-7 tool across national and subnational levels, highlighting potential barriers and facilitators. The qualitative component involved focus group discussions and interviews with Rapid Response Team members leading outbreak response efforts. During the study period, 134 public health events took place, with timeliness data available for 58 events (43%). Among these, detection, notification, and early response targets were met in 36 (62%), 51 (88%), and 50 (86%) events, respectively. All targets were achieved in 28 (48%) of the events. The median time (range) for detection was 6 days (0-149), for notification less than 24 hours (0-4), and for early responses 1 day (1-101). Bottlenecks identified included delayed care-seeking by patients, low awareness or suspicion among private health workers, limited community knowledge, unreported sick or dead poultry, and poor sharing and collaboration within the one-health framework. The 7-1-7 approach provides a clear and practical method for evaluating outbreak responses, pinpointing bottlenecks, and informing the National Action Plan for Health Security (NAPHS) to enhance the next Joint External Evaluation score. This study recommends strengthening early detection through community awareness and public and private healthcare sector engagement, strengthening logistics and coordination, offering regular training, and integrating 7-1-7 indicators into national surveillance systems to improve outbreak preparedness and response.

## Background

Timeliness is a critical determinant of outbreak control. The faster a health system can detect and respond effectively to an outbreak, the more likely it can be brought under control ^1^. The COVID-19 pandemic demonstrated that delays in detection and initiation of response are associated with preventable morbidity, mortality, and socioeconomic -disruption ^2^. Accordingly, strengthening timeliness and adherence to standard operating procedures across core public health functions, such as surveillance, reporting, laboratory confirmation, and risk communication, is foundational to health security ^3^.

 The 7-1-7 target, a timeliness metric, is designed to improve the effectiveness of outbreak management by setting three time-bound milestones: detect suspected events within 7 days of emergence; notify the appropriate public health authority within 1 day of detection; and complete seven core early response actions within 7 days of notification ^4^. The seven early response actions, which include the initial investigation, epidemiological analysis, and other activities, translate the abstract concept of rapid response into tangible and assessable tasks ^5, 6^. The usefulness of 7-1-7 timeliness metrics for improving outbreak detection, notification, and response across multisectoral systems is becoming more widely acknowledged ^7– 9^

Evidence from multiple settings indicates that 7-1-7 is feasible- and operationally useful. In a retrospective, observational analysis of 41 public health events across Brazil, Ethiopia, Liberia, Nigeria, and Uganda, 54% met the 7-day detection target, 71% met the 1-day notification target, and 49% achieved completion of early response actions within 7 days; however, only 27% met all three targets, underscoring opportunities for systematic strengthening ^5^. Reflecting growing momentum, the 7-1-7 approach has been incorporated into regional and global frameworks and is now in routine or pilot use across more than twenty countries, with interest expanding through technical alliances and financing -platforms ^10^. The framework is now widely recognized as a key accountability metric for global health security, with timeliness indicators integrated into the WHO after-action review process ^11^.

Cambodia adopted 7-1-7 in 2023 through the Ministry of Health (MoH). -The Director of the Cambodia Communicable Disease Control Department (CCDC) was identified as the “National 7-1-7 Champion”. All personnel who are involved in response to public health events at national and provincial (sub-national) levels have been trained in the application of 7-1-7. The One Health approach, which multisectoral coordinated action across human, animal, and environmental sectors to prevent, detect, and respond to health threats, is crucial because many public health threats ^12, 13^. Cambodia has established an Inter Ministerial Coordination Committee (IMCC) to address One Health threats; both were endorsed in November 2023. Given Cambodia’s recurrent zoonotic disease, foodborne illnessess and vector-borne risks, cross-border mobility, mixed public–private service provision, and resource constraints, a time-bound, metric-driven approach is needed to prioritize actions, strengthen accountability, and guide continuous improvement towards outbreak responses–particularly within One Health context.

Cambodia’s health security system is overseen by the CCDC, which has served as the National Focal Point for the International Health Regulations (IHR) 2005 since 2007 ^14^. The country is committed to implementing the IHR framework and strengthening its capacity to prevent, detect, and respond to public health emergencies, including outbreaks. As a part of this commitment, Cambodia volunteered to participate in the Joint External Evaluation (JEE) conducted by the IHR in 2016 and 2024. These evaluations were intended to assess the national capacities across core domains of health security. However, the findings highlighted gaps and limitations in capacity that require continued attention and investment ^15, 16^.

Therefore, our study aimed to evaluate the adoption and use of the 7-1-7 target for managing public health events within Cambodia’s health system and to identify contextual factors that facilitated- or hindered achieving the 7-1-7 benchmarks. Specifically, we assessed the progress of 7-1-7 adoption at national and sub-national (provincial/Operational district (OD)) levels, including the attainment of adoption milestones and the actions undertaken to support uptake and integration into existing systems. We examined the system performance against 7-1-7 timeliness targets for events reported between January 2023 and July 2025 by estimating overall performance, comparing performance across event types and provinces, and identifying and categorizing bottlenecks and enablers associated with each target component.

## Methods

### Study design

A sequential explanatory mixed methods design was used in this study. To assess the level of adoption of 7-1-7 timeliness metrics at the national and provincial levels, a cross-sectional study was conducted. A cohort study design was used to assess the use of the 7-1-7 metrics for public health events reported between January 2023 and July 2025. The quantitative study was followed by a descriptive qualitative study comprising focus group discussions (FGDs) and key informant interviews (KIIs) to understand the contextual factors that facilitated or hindered the adoption and use of the 7-1-7 targets.

### General setting

Cambodia is a country located in South Asia, with an estimated population of 17.28 million in 2024 ^17^. Administratively, the country is divided into 25 provinces and 162 districts. Healthcare services are provided through both the public and private sectors. The public health system is organized into three levels: National, Provincial, and Operational District (OD), with health centers functioning under the OD level. At the OD level, health centers are responsible for primary health care delivery and disease surveillance.

### Specific setting

At the national level, under the public health system, services are provided through nine national hospitals and a national laboratory. The national laboratory supports both national hospitals without laboratory capacity and provincial-level health facilities. Provincial Health Departments (PHD) and 25 provincial referral hospitals provide services at the provincial level. At the operational district level, there are 100 referral hospitals and Health Centers (N = 1288). Community-level services are supported by village health support groups (VHSG), established with two volunteers per village. Private health providers operate across all administrative levels and include hospitals, polyclinics (clinics with inpatient facilities), and consultation rooms.

### Management of public health events in Cambodia

Public health events in Cambodia are identified based on national surveillance and response guideline(unpublished). Based on that, there are two surveillance approaches: indicator-based surveillance, where outbreaks are defined to occur when reported cases exceed alert thresholds. The second, event-based surveillance, is based on the occurrence of diseases/events that are reported from communities, health facilities, authorities, or media. Such events are verified by and confirmed by the Rapid Response Team (RRT). Cambodia also utilizes IHR reporting criteria as an instrument to assess and initiate response activities to public health events ^18^. The Emergency Operations Centre (EOC) at the national level, which functions under the CCDC, provides the resources and coordinates management of public health events. The Incident Management System (IMS), a multi-disciplinary group of experts at the EOC, guides the outbreak response in the county based on Standard Operating Procedures (SOP) ^19^. Events reported in the country include emerging/re-emerging diseases, zoonotic diseases, food/water-borne illnesses, vector-borne diseases, and vaccine-preventable diseases.

RRT within the MoH at the national, provincial, district level, and at health care facilities are designated for responding to public health events. At the national level, event-specific RRT function under their respective departments. At the sub-national level, one general RRT team manages all the events. Since 2023, a One Health RRT has been established at the national level with US. Centers for Disease Control and Prevention (US CDC) support to coordinate responses to zoonotic events.

The events are verified and classified by the district/provincial RRT based on type, severity, and complexity, and reports to the national team. Based on the level of risk and severity, RRT deployment and public health measures are initiated. High-risk events are handled by national RRTs, while lower-risk events are managed locally. This classification process consider the likelihood of spread, severity of disease and morbility, potential for disease transmission, and public health implication. All public health events are recorded by PHD RRTs and RRTs from the national level into the event-based surveillance systems known as Cambodia Event Monitoring System (CamEMS) of the CCDC.

**Public health event monitoring system:** The CamEMS is a One Health digital platform managed by the CCDC. The system is used by multiple stakeholders, including the General Department of Animal Health and Production, the Ministry of Agriculture, Forestry, and Fisheries. National and provincial RRTs are responsible for data entry. Once a suspected event is entered into the CamEMS by a provincial RRT, an automated message is sent in the Telegram group alerting the national RRT, resulting in the initiation of a field investigation. The system also allows the capture of other information related to response activities, like the control activities implemented.

### Implementation of 7-1-7

In Cambodia, the 7-1-7 metric was introduced in 2023 with a 7-1-7 sensitization workshop conducted by the CCDC for government departments and partner organizations engaged in surveillance and response. The Director of the CCDC was identified as the “National 7-1-7 Champion” for implementaion 7-1-7 metric and leads coordination mechanisms that support 7-1-7 adoption at all levels. Training was also provided to Cambodian Field Epidemiology Training Program (FETP) graduates and RRT members from all 25 provinces (two members per province). In 2024, a data consolidation workshop was held for RRTs from all provinces and PHD-level decision-makers, facilitated by CCDC in collaboration with the US CDC and Resolve to Save Lives (RTSL). At present, all RRTs at national and provincial levels have been trained in the application of 7-1-7 metrics. The 7-1-7 tool has not been integrated into the existing one health system. The 7-1-7 metric has been implemented as described in Table 1.

**Table 1.** Implementation of 7-1-7 metrics for public health events in Cambodia.

Detection of the outbreak or public health event (First-7)		
**Events**	**Date of emergence**	**Date of detection**
Outbreaks of zoonotic diseases, foodborne diseases, vector-borne diseases, and vaccine-preventable diseases that occur across all 25 provinces and at all levels of health facilities and at communities in Cambodia.	For endemic diseases (e.g. dengue): The date on which a predetermined increase in case or surveillance threshold exceeds over baseline For non-endemic diseases (e.g.A(H5N1)): The date of onset of symptoms in index case or first epidemiologically linked case For rabies, the date of emergence is the date on which the patient reported to the hospital with dog bite For other public health events (e.g. food-poisoning): The date the event first meets the cases definition	The date on which the event is first documented in a paper-based record or reported through event-based surveillance by RRT.
**Notification of the outbreak or public health event (Next-1)**		
**Notifying agency**	**Methods of notification**	**Date of notification**
National/Provincial RRTs	Recording the event into the CamEMS and notifying the CCDC regarding confirmation of the event	The date on which the national RRT notify the CCDC regarding confirmation of the event
**Early Response Actions (Second-7)**		
**Implementing team**	**Date of early response activities**	**Components**
RRTs at national and provincial levels Surveillance officer from national level (CCDC) coordinates the 7 response actions and document dates of completion	Date of early response initiation: Date on which the first of the seven early response actions occurred Date of early responses completion: Date on which all applicable early response actions were completed	1: initiate investigations and deploy team 2: Conduct epidemiological analysis 3: Obtain laboratory confirmation 4: Initiate appropriate management and infection prevention and control (IPC) measures 5: Initiate appropriate public health counter measures 6: Initiate communication activities 7: Establish a coordination mechanism
Enablers and bottlenecks for each of the targets were also determined.		

### Study population

To assess the adoption of 7-1-7, data were collected from RRT representatives who had been trained in 7-1-7 and were involved in public health investigation and response at the national level and all 25 provinces. All the events reported between January 2023 and July 2025 were included to assess the usage of 7-1-7. Among them, the events with data available on the time metrics and bottlenecks/enablers were only included for timeliness and bottleneck/enabler framework analysis. We conducted six FGDs (each with 8-10 participants) and twenty-two KII to capture the qualitative data. The participants for qualitative interviews and discussions were selected purposively based on their knowledge, receipt of training, and their role in 7-1-7 adoption and implementation. The designation and affiliations of the individuals who participated in the FGDs/KIIs are provided in the data repository (Supplementary file 1 and file 2).

### Data collection and data variables

Quantitative component: Data on the adoption of the tool at different levels (national and subnational) were collected using Google Forms, which was developed based on the 7-1-7 adoption checklist modified to adapt to the Cambodia context and consolidated in Microsoft Excel. The data on events were collected as they occurred. In 2023, a designated CCDC epidemiologist liaised with RRTs to record 7-1-7-related data in the 7-1-7 data consolidation Excel spreadsheet. This information was presented and confirmed during the After-Action Review (AAR) meetings. Since 2024, Early Action Reviews (EAR) have been conducted during outbreaks to identify the bottlenecks and enablers, and these have been documented. Key quantitative variables included: event type, date of emergence, date of notification, date of detection, date of completion of early responses, bottlenecks, and enablers.

Qualitative component: Qualitative data were collected using FGDs and KII (Supplemnetary file 3 and file 4). The details of the participants are provided in the data repository (Supplementary file 1 and file 2). FGDs were conducted for public health events reported during 2025, within one week of event notification. The participants were asked about their willingness to participate, and discussions were conducted among willing participants after obtaining written informed consent. The number of FGDs was guided by the saturation of information, and we conducted six FGDs. The FGDs were conducted by qualified medical professionals (Principal investigator (PI) (SM) and co-investigators (SS and HPL) who had been trained in qualitative research. The time duration for FGDs ranged from 60 minutes to seventy-five minutes. The discussions and interviews were conducted at a place and time convenient for the participants, after obtaining informed written consent. To ensure confidentiality, only the participant(s) and members of the research team were present during the interviews. The time duration of the interviews ranged from approximately thirty minutes to forty-five minutes. All discussions and interviews were conducted in the local language (Khmer). Field notes were taken by the PI(SM) to complement the transcripts and capture contextual details. At the end of each discussion/interview, the key points and interpretations were summarized and discussed with the participants to confirm accuracy and allow clarifications. The interviews were conducted until data saturation, leading to 22 interviews. The discussions/interviews were audio recorded after obtaining permission from the participants to ensure the quality of data while preparing the transcript.

### Data management and analysis

Quantitative analysis- Data were retrieved in MS Excel format and analyzed using STATA® (version 16.0, Copyright 1985–2019, StataCorp LLC, College Station, TX, USA). The time duration in days for detection, notification, and completion of responses was calculated with the difference between the respective dates and summarized as median (IQR). The detection time was calculated as the difference between the date of emergence and the date of detection. Notification time was the difference between the date of notification and the detection date. Response completion time was the difference between the date all seven actions were completed and the notification date. The number and proportion of events achieving each 7-1-7 component were calculated for all events and sub-group by type of event, province and time period. The findings are reported using the Strengthening the Reporting of Observational Studies in Epidemiology (STROBE) guidelines ^20^.

Qualitative analysis- The audio recordings were transcribed on the same day of the discussions/interviews and translated into English. Thematic analysis using a hybrid (used both deductive and inductive codes) coding process was followed, and direct quotes were used to highlight the themes. The coding was done using Atlas-ti(trial version 25) ^21^ by researcher (KCP) who systematically reviewed and coded all transcripts. The codes were further reviewed and discussed within the research team to ensure consistency and credibility of interpretation. The codes were then organized into categories and refined iteratively to develop a coding tree in several discussions among the research team, which illustrated the hierarchical relationship between codes, categories, and emerging themes. The findings are reported using the ‘Consolidated Criteria for Reporting Qualitative Research (COREQ) ^22^. The STROBE and COREQ checklists are provided in the data repository.

### Statement on patient and public involvement

Patients and the public were not involved in the planning or conduct of this study.

## Ethical approval and consent to participate

Ethics approval was received from the National Ethics Committee for Health Research, Cambodia (241NECHR). Written informed consent was obtained from all participants involved in the study. All quantitative and qualitative data was anonymized by removing all individual-level identifiers prior to analysis to ensure data privacy and confidentiality. All the data used for the analysis were stored in a password secured computer system.

## Results

### Objective 1- Adoption of 7-1-7 metrics

***Adoption and readiness for 7-1-7 timeliness metrics at the national and subnational levels***

We collected data from 26 informants across national and subnational levels (one informant from the national level and 25 from the subnational level). Among the participants, 17(65.3%) were RRT members/officers, 7 (27%) were RRT managers, and 2(7.7%) were deputy director-level officials. At the national level, the training and adoption were completed with 20 public health staffs being trained on the 7-1-7 tool. At the subnational level, all the provinces (25) had been provided with training and were in the process of adoption, with 70/100(70%) trained public health staffs. In 103 ODs, 42 public health staff had been trained in 26(25.2%) ODs. Government champion and coordination teams were available only at the national level. The 7-1-7 metrics had been integrated into routine public health workflows at the national level, and at the subnational the metrics was integrated only in certain public health events.

Among the subnational representatives, 14(58%) informants perceived that the presence of a 7-1-7 champion would significantly improve adoption in their province. The coordination teams were perceived to greatly improve outcomes by 19 (76%). Among stakeholder roles, coordination leadership (24; 96%) was identified as having the strongest influence, followed by data collection and analysis (21; 84 %). Support from the national level (CCDC) was considered essential by all subnational respondents (100%), with technical support as a frequently reported (100%) need, followed by system support (22; 88%). For stakeholder engagement, the most preferred approach was a combination of all the strategies (13; 52%). Leadership commitment (22; 88%) and availability of funding (18; 72 %) were considered as key factors that strongly influence the integration of the 7-1-7 approach at the subnational level ( Table 2).

**Table 2.** Adoption of the 7-1-7 during 2023–July 2025 in Cambodia at subnational level.

Perceptions among the informants on adoption of 7-1-7	Subnational level (N=25), n (%) ^1^
**Importance of 7-1-7 champion**	
Significantly improves the adoption	14 (56)
Slightly improves the implementation	8 (32)
No effect	3 (12)
**Impact of coordination team**	
Greatly improves outcome	19 (76)
Moderately improves outcomes	4(16)
Significantly reduces outcome	2 (8)
**Stakeholder roles with strong influence in** **7-1-7 adoption ^1^**	
Coordination leadership	24 (96)
Data collection and analysis	21(84)
Planning and financing	18 (72)
Communication and advocacy	11 (44)
Performance improvement	10 (40)
**Type of support needed from national level ^1^**	
Technical Support	25 (100)
System Support	22 (88)
Human Resource Support	11 (44)
Budget	5 (20)
**Effective stakeholder engagement** **approach ^1^**	
One-on-One meeting	9 (36)
Small technical meetings	9 (36)
Large stakeholder conventions	10 (40)
Combination of all approaches	13 (52)
**Factor that strongly influence the** **integration of 7-1-7 metrics into the** **processes ^1^**	
Strong leadership commitment	22 (88)
Stakeholder awareness	16 (64)
Availability of funding	18 (72)
Robust data systems	17 (68)
Clear communication strategies	13 (52)
All this	6 (24)
**Effectiveness of training**	
Highly effective	15 (60)
Moderately effective	10 (40)

^1^ Percentages may exceed 100% as respondents were allowed to select multiple options

***Contextual factors that influence the adoption of 7-1-7***

**Figure 1.**
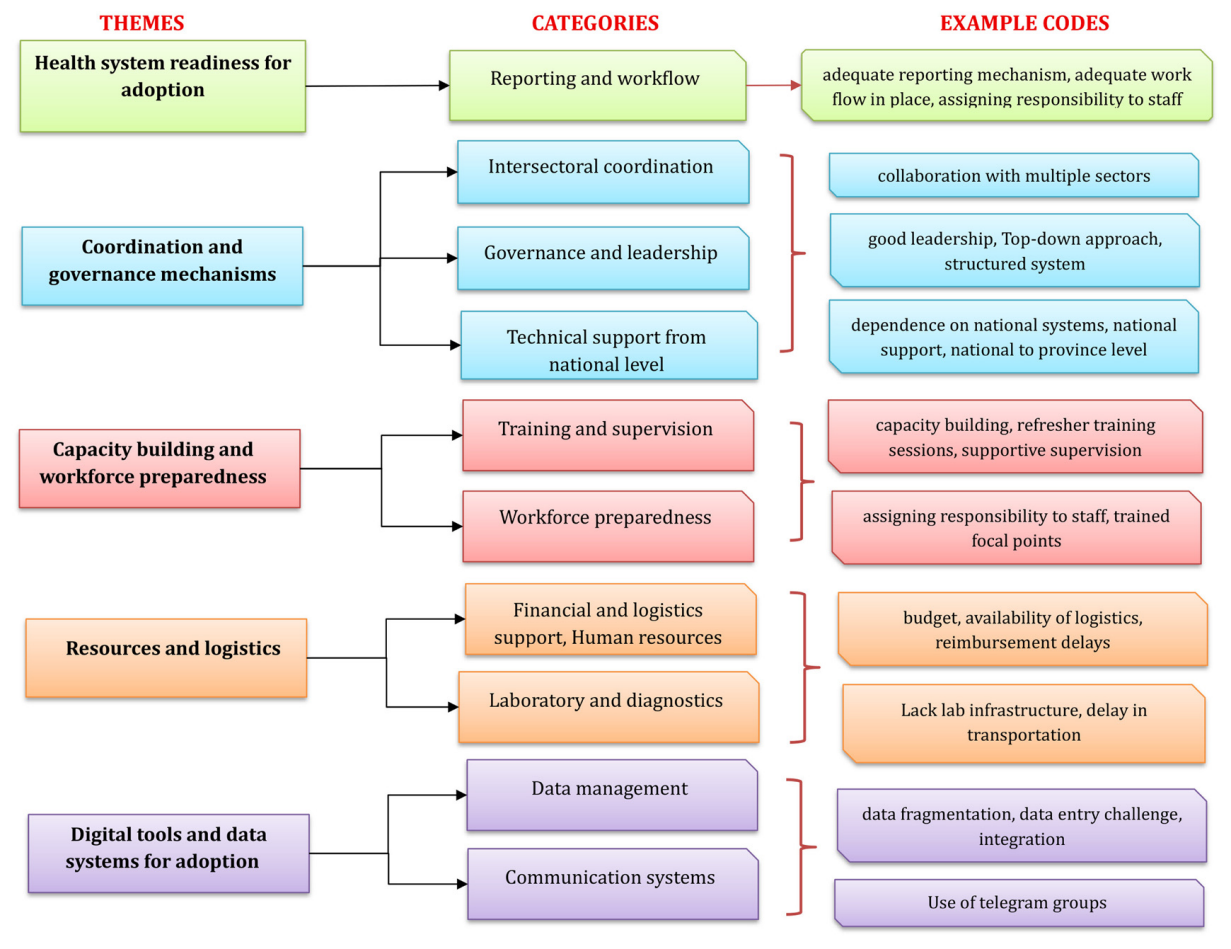
Flowchart showing themes and categories emerged during qualitative analysis on adoption of 7-1-7 in Cambodia. This flowchart presents five major themes, each represented by a distinct color: health system readiness for adoption (green), coordination and governance mechanisms (blue), capacity building and workforce preparedness (red), resources and logistics (orange), and digital tools and data systems for adoption (purple). For each theme, the corresponding categories are displayed in parallel in the center column, and the example codes derived from qualitative interviews are shown in the right-most column. The diagram illustrates how individual codes map onto broader categories and themes identified through thematic analysis.

Thematic analysis of qualitative data collected using the KII technique from 22 participants to understand the contextual factors that facilitate or hinder adoption resulted in the emergence of five themes and eleven categories from 162 codes ( Figure 1)

**(i)Health system readiness for adoption**

Most participants opined that the adoption of 7-1-7 at the subnational level is facilitated by the established workflow system and the readiness of public health staff to adopt the tool.

 “ *It seems no issue related to this(adoption) because we have RRT from either ministry, PHD, OD, and other ministries to work on this, and the work is quickly responded to. We work quickly, therefore the national staff are also quick to respond as well, for instance, if the event happened at night, in the morning the national staff arrive and support the next day and work until the case is closed*.” (KII17)

**(ii)Coordination and governance mechanisms**

All participants believe that coordination across PHDs, ODs, hospitals, and health centers is essential for adoption, but it is often uneven. In most of the events, RRTs from different levels worked quickly and effectively; however, at certain times, slow information flow and a lack of coordination within the health sector created hindrances.

*“Coordination between PHD, OD, hospital, and health centers is sometimes slow, and multi-sectoral collaboration remains weak.”* (KII19)

The collaboration between multiple departments, such as the animal sector, fisheries, the environment department, and village health support groups, is often lacking in many provinces. Many participants felt that targeted awareness campaigns, orientation sessions, and regular communication are needed to build a shared understanding among stakeholders to ensure active participation in adopting and achieving the 7-1-7 goals.

“…a *s in the case of dengue fever, in the past, **we** **only** went to health centers, communities, and villages, and others (other departments) did not fully participate. However, if there is someone from the national level with us, everyone works together to strengthen the local community, the process goes smoothly..*.” (KII 20)

All the participants opined that good leadership support plays a key role in the adoption of the tool in their province. Good leadership support has facilitated the adoption and effective application of the tool. However, the majority commented on the need for national support in terms of guidelines and recommendations to effectively adopt and utilize the tool.

**(iii)Capacity building and workforce preparedness**

Although subnational-level staff are provided with training, many participants reported a lack of confidence in implementing the tool and expressed a need for refresher training. The trainers at the national level are reported to be approachable; however, participants opined that the lack of follow-up from the national level has resulted in losing track of the procedures. The majority of the participants believe that successful integration will require regular training and supportive supervision at regular intervals.

*“There should be an invitation to all relevant subnational staff for the discussions and orientation meeting to identify the bottleneck/solution. We would like to request technical support from the national level to the subnational level regularly, because if we do not apply this tool regularly, we may forget about it. There must be regular follow-up questions on whether this tool is well implemented and reported on time or not. Also, if there is any update on this tool, the refresher training should be provided to all relevant staff*.” (KII18)

**(iv)Resources and finances**

All the participants believe that the adoption is affected when resources and logistics are insufficient. Several provinces lack the necessary laboratory infrastructure, and some depend on the national laboratory. Even the national laboratory in the country is not well-equipped to conduct all the required laboratory investigations.

*“…For example, a case of food poisoning at Koh Nhek, the provincial laboratory has been trying its best for the test result, but couldn’t. We then sent the sample to Phnom Penh (the national laboratory), but they still couldn’t confirm. So, this meant that not only provincial level, but also national laboratories have their limitations…” (KII 19)*

In addition, the lack of sufficient a number of trained RRT members has resulted in the overburdening of existing staff with work required to respond to the events. Shortages in budget, staffing, and support for routine activities continue to be common barriers at the subnational level.

“… *We haven’t done any training since we finished the ToT training in Siem Reap. Why? This is because we do not have a budget package for this.*..” (KII 12)

The majority of the participants have also commented on the need for technical materials like guidelines, checklists, and SOPs in local languages for better understanding and application of the tool.

**(v)Digital tools and data systems for adoption**

All of the participants opined that digital systems can make adoption and implementation easier by reducing fragmentation and improving monitoring.

*“…There are challenges, such as we do not yet use the system because we at the moment still use the paper-based...we do not know who to ask for help on this…”* (KII18)

All of the participants commented that the existing systems (CamEMS) in the country are not aligned to capture the data needed for 7-1-7, and several diseases/events have different tools. In the case of MIS (Malaria Surveillance System), it is based on 1-3-7. Also, the CamEMS does not capture all required details, and staff struggle with paper-based or unfamiliar apps. The majority of the participants commented that if the 7-1-7 indicators are incorporated into the routine data reporting system and supported by dashboard visualizations, it would significantly improve their ability to monitor delays and identify bottlenecks. 

*“….. in CamEMS, can we integrate this tool into CamEMS? Can we modify and allow report entry, such as date or line listing, in this (CamEMS)? If we get this system integrated as one health, then it will be very efficient; in a few clicks, we will be able to see it all.”* (KII16)

### Objective 2- Use of 7-1-7 metrics

**Figure 2.**
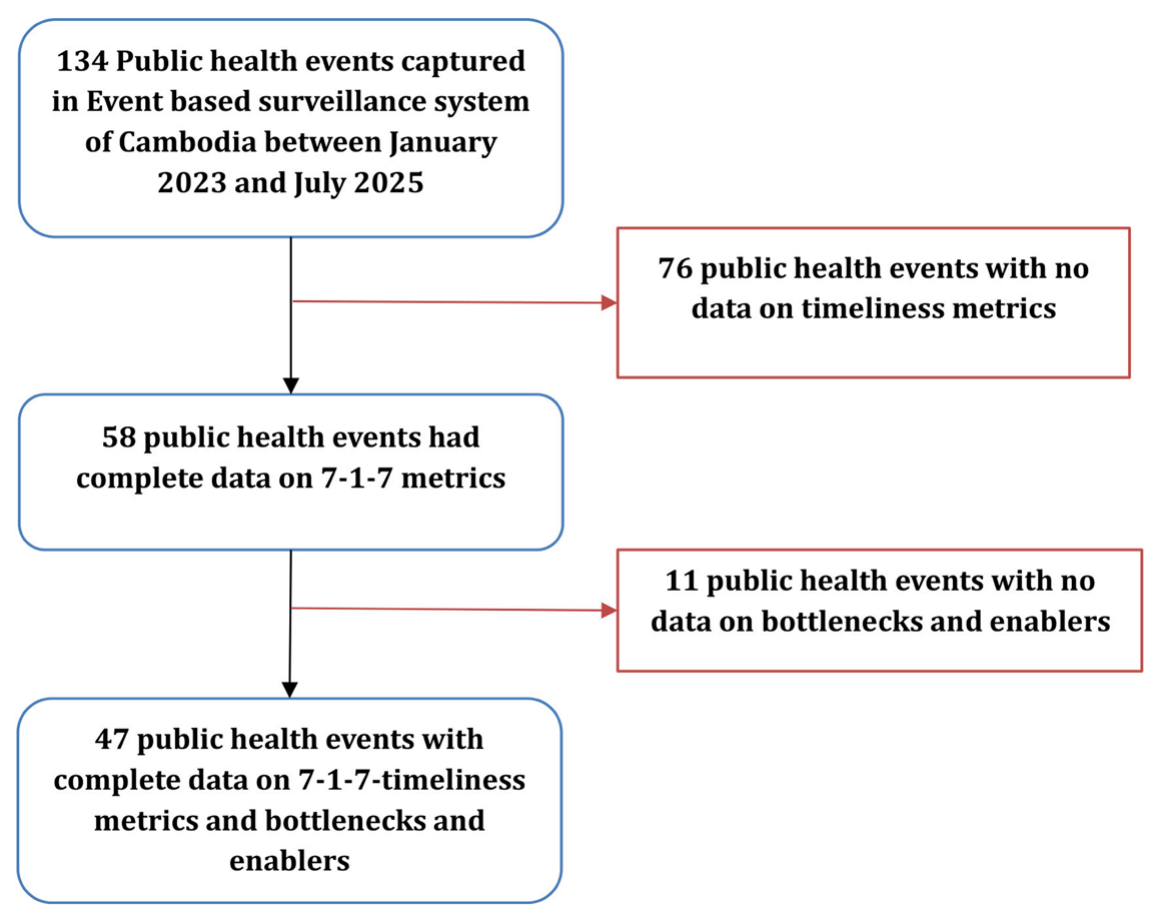
Number of public health events captured through Event-Based Surveillance in Cambodia, January 2023–July 2025. Figure 2 presents the number of public health events captured through the national event-based surveillance system and the subset with complete data for 7-1-7 timeliness metrics, bottlenecks, and enablers. The figure shows sequential exclusions due to missing timeliness data or missing qualitative information.

During the study period, 134 public health events have been reported in the country. Among them, 58(43%) have time metric data captured and were used in our analysis. For 47(35%) events, bottlenecks and enablers were captured using the taxonomy ( Figure 2).

***Overall use of 7-1-7 timeliness metrics***

Among the 58 public health events reported between January 2023 and July 2025, 26 (45%) events were respiratory illnesses (A/H5N1, flu-like illness cluster). Foodborne illnesses constituted 12 events (21%), vector-borne diseases (malaria and dengue) accounted for 7 events (12%), and 11(19%) events were zoonotic (rabies, mpox). Vaccine-preventable diseases (measles) accounted for 2 events (3%) ( Table 3).

**Table 3.** Median timeliness and proportion of events meeting 7-1-7 targets in Cambodia, January 2023- July 2025.

Public health event	Number of Public Health Events(N)	Detection (target=7 days)		Notification (target=1 day)		Completed early response (target= 7 days)		7-1-7 met target (all targets met) (n ^1^/N) (%)
		Median days (range)	Met target (n ^1^/N) (%)	Median ^2^ days (range)	Met target (n ^1^/N) (%)	Median days (range)	Met target (n ^1^/N) (%)	
**ALL**	**58 (100)**	**6 (0-149)**	**36 (62)**	**0 (0-4)**	**51 (88)**	**1 (1-101)**	**50 (86)**	**28 (48)**
A/H5N1	25 (43)	6.5 (3-12)	15 (60)	0 (0-3)	21(84)	1 (1-27)	22 (88)	12 (48)
Mpox	6 (10)	12.5 (9-55)	0 (0)	0 (0-2)	5 (83)	6.5 (1-12)	5 (83)	0 (0)
Rabies ^3^	5 (9)	56 (0-149)	2 (40)	0 (0-0)	5 (100)	1 (0-1)	5 (100)	2 (40)
Malaria	5 (9)	14 (7-28)	2 (40)	0 (0-0)	5 (100)	6.5 (2-11)	2 (40)	1 (20)
Dengue	2 (3)	1.5 (0-3)	2 (100)	2 (0-4)	1 (50)	51 (1-101)	1 (50)	0 (0)
Measles	2 (3)	4.5 (3-6)	2 (100)	0 (0-0)	2 (100)	8.5 (2-15)	1 (50)	1 (50)
Respiratory illness (flu cluster)	1 (2)	0 (0-0)	1 (100)	3 (3-3)	0 (0)	0 (0-0)	1 (100)	0 (0)
Foodborne Illnesses ^4^	12 (21)	0 (0-2)	12 (100)	0 (0-1)	12 (100)	2 (0-7)	12 (100)	12 (100)

^1^ number of events that met the specific target, ^2^ Median days of 0 equals less than 24 hours, ^3^ In rabies, the date of emergence is the date at which the patient reported with complaints of dog bite in the health facility. ^4^ foodborne poisoning (pufferfish Toxin=4, methanol Poisoning=2, unknown sources =6)

The median (IQR) time to detection was 6 days (2-9), ranging from less than 24 hours to 149 days in rabies events. Approximately 62% (36) of the events met the target of detecting the outbreak within 7 days. The median (IQR) time to notification was less than 24 hours (0-1), with a maximum of 4 days seen in dengue events. Among the reported events, 88% (51) met the target of notifying within 24 hours. The median (IQR) time to complete the seven early response actions was 1 day (0-3), with a range of 1 to 101 days. A total of 86% (50) of events met the target of completing early response actions within 7 days. Overall, 48% (28/58) of events met all three 7-1-7 targets. Performance varied by type of public health event. Full compliance with all 7-1-7 targets was observed for foodborne illnesses of unknown source (6/6), pufferfish toxin poisoning (4/4), and methanol poisoning (2/2). In vector-borne diseases, none of the dengue (0/2) events met all targets, and only one of five malaria events (1/5; 20%) achieved full compliance. 

***Use of 7-1-7 timeliness metrics across provinces and time periods***

Across provinces, Takeo province showed strong performance, with 3 out of 4(75%) achieving all three targets. Phnom Penh recorded the highest number of events, but none met the detection target (0/7), although 6(85.7%) events met notification and all achieved a timely response ( Table 4).

**Table 4.** Timeliness and proportion of events meeting 7-1-7 targets across Cambodia’s provinces, January 2023- July 2025.

Provinces	Number of Public Health Events N(%)	Detection (target=7 days)		Notification (target=1 day)		Completed Early Response (target= 7 days)		7-1-7 – all targets met (n ^1^/N) (%)
		Median days (range)	Met target (n ^1^/N) (%)	Median ^2^ days(range)	Met target (n ^1^/N) (%)	Median days (range)	Met target (n ^1^/N) (%)	
**ALL**	**58 (100)**	**6 (0-149)**	**36 (62)**	**0 (0-4)**	**51 (88)**	**1 (1-101)**	**50 (86)**	**28 (48)**
Phnom Penh	7 (12)	14 (9-56)	0 (0)	0 (0-2)	6 (86)	0 (1-12)	6 (86)	0 (0)
Kampong Speu	6 (10)	8 (8-28)	3 (50)	0 (0-0)	6 (100)	7 (1-14)	3 (50)	2 (33)
Prey Veng	5 (9)	7 (6-10)	3 (60)	2 (0-3)	2 (40)	1 (1-25)	4 (80)	1 (20)
Svay Rieng	5 (9)	6 (6-6)	4 (80)	0 (0-1)	5 (100)	3 (0-27)	4 (80)	3 (60)
Siem Reap	4 (7)	6 (1-9)	2 (50)	0 (0-1)	4 (100)	1 (1-23)	4 (100)	2 (50)
Takeo	4 (7)	4.5(2-9)	3 (75)	0 (0-0)	4 (100)	1 (0-2)	4 (100)	3 (75)
Kampot	3 (5)	6 (4-10)	2 (67)	1 (0-1)	3 (100)	1 (0-1)	3 (100)	2 (60)
Kampong Cham	3 (5)	0 (0-8)	2 (67)	0 (0-0)	3 (100)	3 (2-101)	2 (60)	1 (30)
Ratanakiri	3 (5)	6 (0-146)	2 (67)	0 (0.8-2.3)	3 (100)	1 (1-2)	3 (100)	2 (60)
Kandal	2 (3)	6 (0-12)	1 (50)	1.5(0-3)	1 (50)	1 (1-2)	2 (100)	1 (50)
Koh Kong	2 (3)	0 (0-0)	2 (100)	2 (1-3)	1 (50)	1 (1-3)	2 (100)	1 (50)
Kratie	2 (3)	7.5 (6-9)	1 (50)	0 (0-0)	2 (100)	1 (1-1)	1 (100)	1 (50)
Province ^3^	3 (5)	3 (3-22)	2 (60)	0 (0-4)	2 (60)	2 (1-15)	2 (60)	0 (0)
Provinces ^4^	9 (16)	1 (0-6)	9 (100)	0 (0-1)	9 (100)	2 (0-7)	9 (100)	9 (100)

^1^number of events that met the specific target, ^2^Median days of 0 equals less than 24 hours ^3^Three provinces, each with one event with all targets of 7-1-7 not met; Kep (N=1), Pailin (N=1), Preah Sihanouk (N=1), ^4^Nine provinces combined, each province with one event with all targets of 7-1-7 met ;Kampong Thom (N=4), Kampong Chhnang(N=1), Mondulkiri (N=1), Oddor Meanchey (N=1), Pursat (N=1), Tbong Khmum(N=1)

Across the time period, the proportion of events detected within 7 days was 57% (4/8) in 2023, 63% (17/27) in 2024, and 35% (8/23) in 2025 from January to July. The median number of days for detection reduced from 8 in 2023 to 5 in 2025. Notification within one day remained high throughout the period, with 100% of events notified in both 2023 and 2025. Completion of early response actions within 7 days improved over time, from 50% in 2023 to 100% in 2025 ( Table 5).

**Table 5.** Timeliness and proportion of events meeting 7-1-7 targets in Cambodia during January 2023- July 2025.

Year	Number of Public Health Events(N)	Detection (target=7 days)		Notification (target=1 day)		Completed Early Response (target= 7 days)		7-1-7 – all targets met n ^1^/N (%)
		Median days (range)	Met target n ^1^/N (%)	Median ^2^ days(range)	Met target n ^1^/N (%)	Median days (range)	Met target n ^1^/N (%)	
Total	58 (100)	6 (0-149)	36 (62)	0 (0-4)	51 (88)	1 (1-101)	50 (86)	28 (48)
2023(January -December)	8 (14)	8 (3–32)	4 (57)	0 (0–2)	8 (100)	6.5 (0–27)	4 (50)	1 (13)
2024(January -December)	27 (46)	6 (0–55)	17 (63)	0 (0–4)	21 (78)	2 (0–101)	23 (85)	12 (44)
2025(January -July)	23 (40)	5 (0–149)	8 (35)	0 (0–1)	23 (100)	1 (1–3)	23 (100)	5 (22)

^1^number of events that met the specific target ^2^Median days of 0 equals less than 24 hours

***Bottlenecks and enablers***

A total of 168 bottlenecks were identified across 47 public health events. These were distributed across detection (86; 62%), notification (14; 10%), and response (68; 47%). The most commonly reported barriers to detection included delayed care seeking (24; 27%) and low awareness or clinical suspicion among private health workers (19; 22%). Bottlenecks in notification were primarily due to the absence of clinical surveillance focal points (4; 28%) and delayed sample transportation (3; 21%). For completion of early response, frequent challenges were low community knowledge or trust (11; 17%), lack of multisectoral response teams (8; 12%), and limited One Health information sharing (7; 11%) ( Table 6).

**Table 6.** Bottleneck identified from AARs/EARs of public health events (n=47), January 2023-July 2025.

Bottleneck categories	Detection (N=86) n (%)	Notification (N=14) n (%)	Response (N=68) n (%)	Total (N=168) n (%)
**Limited knowledge, awareness, and reporting**				
Low awareness or clinical suspicion by private health workers	19 (22)	1 (7)	2 (3)	22 (16)
Lack of community knowledge (regarding exposure)	13 (15)	0	4 (6)	17 (12)
Low community knowledge or trust	0	1 (7)	11 (17)	12 (9)
Presence of ill or dead poultry not reported in the community	15 (17)	0	0	15 (11)
Fear of culling poultry upon event detection	1 (1)	0	1 (1)	2 (1)
Community unreported a exposure	1 (1)	0	0	1 (0.07)
No surveillance repotting in private care facilities	1 (1)	0	0	1 (0.07)
**Health-seeking behavior and access barriers**				
Delayed care-seeking by patient/seeking-care local private clinics (3)	24 (27)	0	0	24 (17)
Poverty prevents people from seeking care	5 (6)	0	0	5 (3)
Limited access to health facilities (long distance)	0	0	2 (3)	2 (1)
School located in a remote area	1 (1)	0	0	1 (0.07)
**Coordination and information sharing gaps**				
Lack of one health information sharing/collaboration	1 (1)	1 (7)	7 (11)	9 (6)
Limited collaboration from the environmental sector	0	0	2 (3)	2 (1)
Lack of multisectoral/disciplinary response teams	0	0	8 (12)	8 (7)
**Detection, diagnostics, and laboratory capacity**				
Rapid tests are unavailable at health center level	1 (1)	0	0	1 (0.07)
Rapid tests negative at the hospital	1 (1)	0	0	1 (0.07)
Delayed sample collection	1 (1)	2 (14)	0	4 (3)
Delay sample transportation	1 (1)	3 (21)	1 (1)	5 (4)
Delayed laboratory confirmation	0	2 (14)	4 (6)	6 (5)
No laboratory sample collection	0	0	1 (1)	1 (0.07)
Limited laboratory capacity at the provincial level	0	0	1 (1)	1 (0.07)
Limited laboratory testing for tetrodotoxin	0	0	3 (5)	3 (2)
Shortage of test material and sample collection supplies	0	0	3 (5)	3 (2)
New or unexpected pathogen	0	0	3 (5)	3 (2)
Limited clinical case management capacity	0	0	1 (1)	1 (0.07)
**Surveillance and notification delays/resource**				
Lack of clinical surveillance focal point/ delayed of reporting	0	4 (28)	0	4 (3)
Limited funds available at the provincial level	0	0	1 (1)	1 (0.07)
**Environmental and risk communication gaps**				
Poor waste management, and sanitation in the community	1 (1)	0	2 (3)	3 (2)
Risk communication was not conducted	0	0	5 (7)	5 (4)
Limited of public health countermeasures in affected communities (vaccine availability at the health center)	0	0	6 (9)	6 (5)

AAR- After Action Reviews, EAR- Early Action Review

A total of 226 enablers were identified. These were noted in the response (94; 42%), notification (77; 34%), and detection (55;24%) phases. In the detection phase, the most reported enablers were strong clinical suspicion or awareness of case definitions (13;24%) and active or sentinel surveillance (14;25%). Notification was facilitated by strong reporting lines for surveillance (33;43%) and availability of resources for rapid mobilization (16; 21%). During response, key enablers included coordinated response mechanisms (28;30%) and the availability of medical or supplier countermeasures (15;16%) ( Table 7).

**Table 7.** Enablers identified from AARs/EARs of public health events (n=47), January 2023- July 2025.

Enabler categories	Detection (N=55) n (%)	Notification (N=77) n (%)	Response (N=94) n (%)	Total (N=226) n (%)
**Community awareness and engagement**				
Community aware of ill/died poultry and reported to authorities	1(2)	0	0	1 (0.4)
Community knowledge or trust of public health system	1(2)	1 (1)	2 (2)	4 (2)
Risk communication and community engagement	0	0	4 (4)	4 (2)
**Clinical capacity and vigilance**				
Strong clinical suspicion or awareness of case definition by health workers	13 (24)	1 (1)	1 (1)	15 (7)
Promptly seeking-care by patients	2 (4)	0	0	2 (0.9)
Case management capacity	0	0	2 (2)	2 (0.9)
**Surveillance and reporting**				
Active or sentinel surveillance in place	14 (25)	0	7 (7)	21 (9)
Strong reporting lines for public health surveillance	2 (4)	33 (43)	0	35 (16)
Coordination and communication between clinical and public health systems	10 (18)	7 (9)	4 (4)	21 (9)
**Laboratory and specimen handling**				
Laboratory diagnostic capability	2 (4)	2(3)	0	4 (2)
Promptly specimen collection	1(2)	0	0	1 (0.4)
Specimen transportation	1(2)	0	0	1 (0.4)
**Response resources and logistics**				
Availability of PEP at PEP centers	2 (4)	0	0	2 (0.9)
Availability of medical/supplier countermeasures (Vaccination/Vector/education material)	0	0	15 (16)	15 (7)
Availability of resources for response initiation or rapid resource mobilization	5 (9)	16 (21)	4 (4)	25 (11)
**Coordination and response mechanisms**				
Rapid coordinated response mechanism in place	0	10 (13)	28 (30)	38 (17)
One health information sharing/collaboration	1(2)	7 (9)	3 (3)	11 (5)
Excellent cooperation between health staff and local authorities	0	0	10 (11)	10 (4)
Rapid response team deployment mechanism in place (One Health)	0	0	7 (7)	7 (3)
Multi-sector and multi-stakeholder collaboration (including partners)	0	0	7 (7)	7 (3)

AAR- After Action Reviews, EAR- Early Action Review

***Qualitative synthesis of enablers and bottlenecks for the use of 7-1-7 metrics***

**Figure 3.**
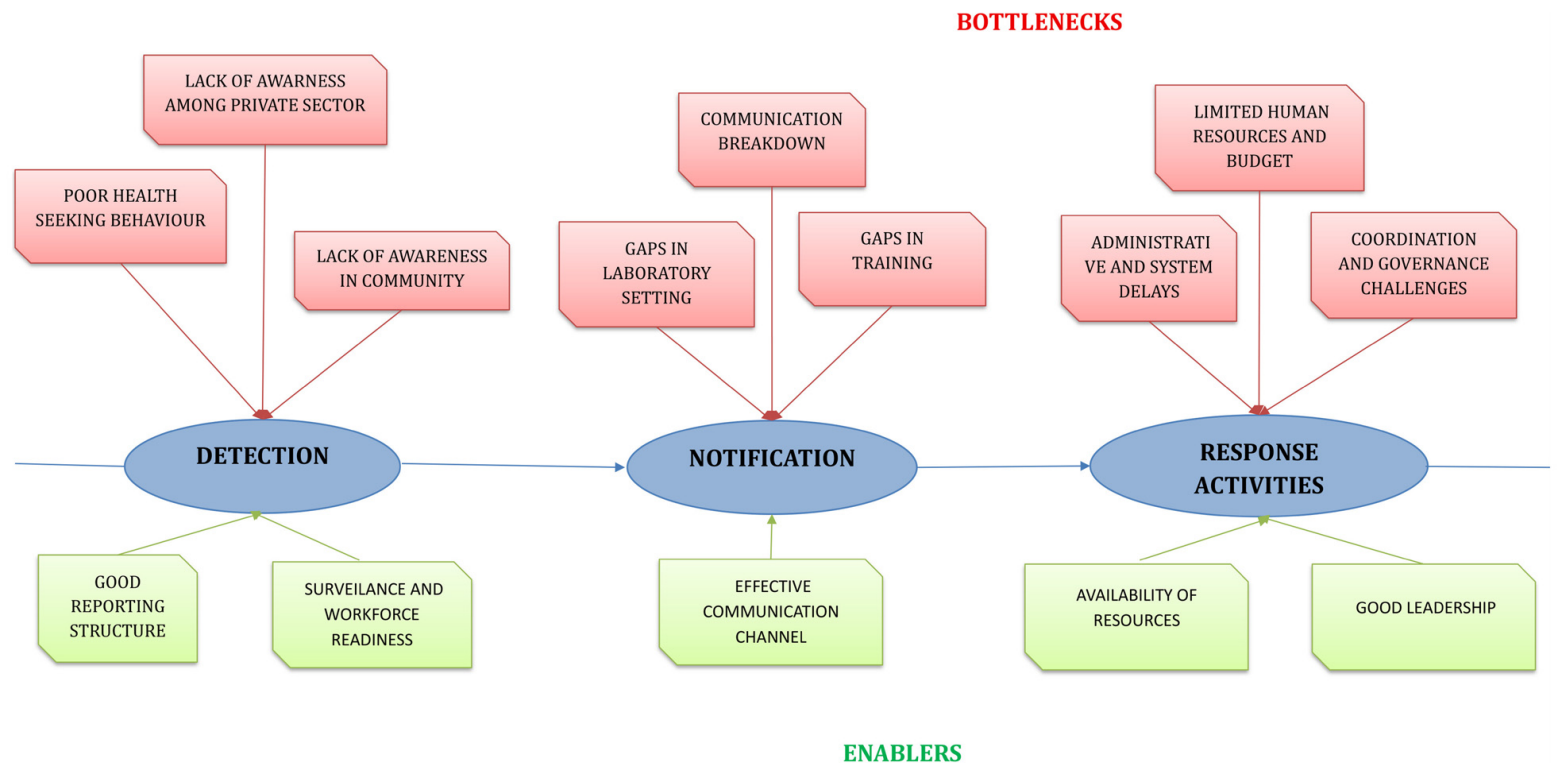
Bottlenecks and Enablers identified in qualitative analysis for detection, notification, and response in using 7-1-7. This diagram summarizes the bottlenecks (in red) and enablers (in green) identified through qualitative analysis across the three steps of the 7-1-7 pathway; detection, notification, and response activities.

The qualitative analysis of six FGDs resulted in the emergence of five themes and 12 categories from 99 codes. Factors such as team coordination, intersectoral collaboration, and availability of human resources emerged as enablers in some settings, while in others they acted as bottlenecks ( Table 8). The bottleneck and enabler categories that emerged during the qualitative analysis specific to each target are provided in Figure 3.

**Table 8.** Themes and categories emerged during qualitative analysis on use of 7-1-7 in Cambodia.

Theme	Categories	Examples of codes
**Community beliefs and health seeking behaviors affects detection**	Low risk perception and norms	normalization of poultry deaths, adhering to traditional treatment, under reporting fearing loss of income
	Health care seeking behavior of people	prefer private providers, private sector lack of vigilance, public hospital last resort, delay in seeking care due to loss of wages, misdiagnosis
**Laboratory and data sharing influence notification**	Laboratory capacity and workflow	adequate lab infrastructure, delay in lab testing, following SARI workflow, lack of urgent marking
	Data transfer and communication breakdown	lack of communication in urgency, delay in result release, multiple telegram groups
**Coordination and governance structures influence response**	Coordination and governance	Top-down approach, from national to provincial, limited intersectoral coordination, lack of adherence to reporting, absence of wildlife/environment teams
	Communication & Reporting Structure	telegram group for RRT, coordinated activity from national to province
**7-1-7 tool implementation anchors on awareness, capacity building and team motivation**	Awareness and knowledge	Community awareness, knowledge of public health staff, awareness of case definitions
	Training	capacity building among PH staff
	Good leadership and workforce Readiness	trained on 7-1-7, encouraging community engagement, team motivation, prompt response
**Operational sustainability requires resources, digital system and policy**	Resource and logistics availability	adequate resources, availability of logistics
	Administrative and systemic capacity	administrative approvals, Government funding for response
	Resource & Policy	policy for compensation, educational material in local language, SOPs for regular refresher training, SOPs for regular supervision

**(i) Community beliefs and health-seeking behaviors affect detection**

All of the participants opined that in the public health sector, a mechanism has been established for communication and transfer of information, which enables detection without delay. The majority of the participants commented that the clinicians in the public sector are experienced in detecting the diseases as per case definitions and promptly inform the authorities.

However, at the community level, individuals tend to adhere to traditional treatment or prefer private hospitals due to ease of access and lack of waiting time. This delays the detection, as the private hospital clinicians either misdiagnose or do not report to the authorities. The individuals also delay seeking health care with over-the-counter medications. The poultry deaths are often underreported due to loss of income or lack of compensation for culling, and even in certain cases, deaths among the poultry are considered normal by the community.

“ *Hmmm...! Usually, patients or families often visit private health facilities and bypass public health facilities; they tend to visit private healthcare providers near their home, and in this case, it was the same pattern, and private healthcare providers misdiagnosed the illness as a common cold.*” FGD1P5, Event Type: H5N1 (Avian Influenza)

**(ii) Laboratory and data sharing influence notification**

Participants in all FGDs commented that, for the notification of events within one day, the laboratory infrastructure and flow of information play a critical role.

“ *NIPH (sentinel lab for influenza surveillance) is immediately informed of H5N1 cases, and samples are sent. Once the laboratory diagnosis is confirmed, the results are shared with the director of CCDC and the clinician. CCDC shares the information through phone and Telegram groups, with national and subnational Rapid Response Teams (RRTs) and relevant stakeholders to take action following the guideline.”* FGD1 P8, Event Type: H5N1 (Avian Influenza)

The delay in sending the sample, the lack of laboratory infrastructure at certain provincial levels, and dependence on the national laboratory affect timely laboratory diagnosis and notification. The lack of communicating the urgency to the laboratory staff at times has resulted in the delay of laboratory confirmation. Once the confirmed results are obtained from the laboratory, the workflow is established for prompt notification.

“ *Another challenge is the process of confirming lab results. Sometimes, there are delays in receiving laboratory confirmation from the central level, which affects how quickly we can notify and take action.”* FGD2P1, Event type: H5N1 (Avian Influenza)

**(iii) Coordination and governance structures influence response**

The majority of the participants opined that effective response depends not only on coordination between health, animal, and other sectors, but also on clear governance structures that define authority, decision-making, and accountability. Where these systems are strong, joint investigation teams and reporting lines work smoothly, but weak intersectoral collaboration, unclear responsibilities, and lack of specialized teams delay and limit the responses.

*“… as soon as the event was suspected, we immediately contacted the foodborne disease focal person to initiate specimen collection. We also mobilized joint investigation teams right away. The reporting processes are well understood among the teams and were followed as expected. Our established referral system further supported efficient case management and escalation when needed*.” FGD3P4, Event type- Suspected Khmer noodle poisoning

**(iv) 7-1-7 tool implementation anchors on awareness, capacity building, and team motivation**

All of the participants were of the opinion that, in Cambodia, the implementation of 7-1-7 is influenced largely by awareness among the community, knowledge among the staff, training received, and good leadership that motivates the team members. Awareness among the community on preventive measures of public health events and the need to alert the health authority enables detection on time. Also, communities with better knowledge are more likely to report unusual events like rabies. In addition, the knowledge of public health staff is central to the use of the 7-1-7 tool. Trained and supervised health staff can apply case definitions quickly and accurately.

“… *I also noticed that good leadership made a difference. It motivated local staff to act quickly and to follow procedures. When there’s strong guidance from the top, things move more efficiently on the ground*” -FGD6P7, Event Type: H5N1 (Avian Influenza)

**(v) Operational sustainability requires resources, digital systems, and policy**

All of the participants opined that the sustainable use of the 7-1-7 metrics depends on the availability of adequate funding, human resources and logistics, and supportive policies.

“ *Our primary challenge is securing funding support at the provincial level for outbreak investigation and response. In some cases, responders, including RRT members, had to spend their own money in advance to implement early actions….*” FGD4P4, Event type: food-borne illness (Puffer fish poisoning)

In addition to funding, timely administrative approvals, mechanisms for regular supervision, and guidelines for refresher training at regular intervals are considered important. In situations with limited staff and lack of trained capacity, the activities didn’t provide outcomes as intended. In addition, delays in reimbursements and limited use of digital platforms weaken the use of the 7-1-7 tool and affect its sustainability.

“ *We had no structured recommendations coming out of the response. No formal risk assessment was done. The investigation was limited to the reported cluster. Across all levels, especially at the OD and health Center, we lacked enough staff to do a full response*.” FGD5P7, Event type: Mushroom poisoning

## Discussion

This mixed-method study assessed the adoption and use of the 7-1-7 timeliness framework within Cambodia’s public health system. The study has four key findings. First, the 7-1-7 framework has been fully adopted at the national level, and at the subnational level, all provinces have undergone training, with adoption in progress. Second, several factors were found to influence adoption, including health system readiness, availability of a coordination team, visible leadership or a champion, regular capacity-building activities, adequate resources and financing, and digital tools and data systems. Third, of the 58 analyzable events reported during the study period, targets were attained for notification (88%) and response in the majority of events (86%) with a median time of less than 24 hours and one day, respectively; detection was comparatively less well achieved (62%) with a median time of 6 days. For 28(48%) events, all targets were met. Finally, among the bottlenecks identified, 62% were for detection, with delayed care seeking and low awareness among private health workers as major contributing factors. The qualitative findings were consistent with the quantitative results and provided contextual explanations. 

Early detection, timely notification, and rapid response are critical for outbreak control ^8, 9, 12^, as they enable swift action to interrupt transmission, reduce morbidity and mortality, and prevent localized events from escalating into larger epidemics ^23^. The median number of days for each target observed in Cambodia were comparable to findings from countries such as Kenya ^24^. However, in contrast to many settings where early response actions are delayed ^5^, Cambodia achieved rapid completion of response activities within a median(IQR) time of 1(0–3) days. This achievement is likely attributable to the experience gained during the COVID-19 pandemic and the continued efforts towards strengthening of the public health sector in managing any health emergencies in the nation ^25^. During the pandemic, Cambodia developed substantial capacity, including approximately 3,000 RRT and 285 public health officers trained in Applied Epidemiology Training and the Field Epidemiology Training Program. These trained professionals, deployed across national and subnational levels, contributed significantly to ensuring rapid responses to public health events ^26^.

On the other hand, detection remains the weakest target, with several bottlenecks, including reliance on private and informal or traditional care pathways, particularly in events such as mpox, rabies, and malaria, with a median(range)days of 12.5(9–55), 56(0–149), and 14(7–28) respectively for detection. Another challenge was the low awareness and limited clinical suspicion among private health workers, especially in the case of A/H5N1, which contributed to 45% (26) of the total analyzable events with a median(range) time of detection of 6.5(3–12) days. The lack of community awareness and fear of economic loss due to culling of poultry were other bottlenecks for timely detection in these events. These bottlenecks were similar to the challenges leading to delayed detection and reporting in several low- and middle-income countries ^27, 28^. In the event of A/H5N1, to bring a positive behavior change among the community, a range of regulatory, fiscal, and nudging policies can be utilized ^29^ which might help in improving the reporting to the authorities and thereby the timely detection of the events. 

Our study has several strengths: first, it included events reported over three years from all the provinces, using national event-based surveillance data, representing a real-world performance. Second, the use of a sequential explanatory mixed-methods design allowed us to contextualize the quantitative findings with the perspectives of the people, strengthening both interpretability and applicability. Third, by disaggregating results according to event type, province, and time period, the study provides granular insights into where and when timeliness targets were achieved or missed, thereby providing actionable directions for targeted improvements. Finally, the study adhered to established reporting standards, including STROBE ^20^ for quantitative analyses and COREQ ^22^ for qualitative analyses, enhancing methodological rigor and transparency.

The study has a few limitations. Complete timeliness data were available for only 58 of 134 events. If better-organized responses were more likely to have complete data, this might have introduced selection bias, potentially overestimating system performance. In addition, certain event types and provinces with fewer numbers of events appeared to have achieved a higher proportion of timeliness targets, a finding that requires validation with a higher number of events before concluding. Finally, subnational adoption of the 7-1-7 framework and integration into the system were ongoing during the study period; hence, our study findings represent a transitional stage rather than performance under full implementation. 

The study has several policy implications. First and foremost is the need to prioritize early detection through community and private-sector engagement. The biggest gap in 7-1-7 performance in Cambodia was in the timely detection of events. Policy efforts should focus on improving health-seeking behavior in communities through targeted risk communication and public awareness campaigns. In parallel, private-sector clinics and pharmacies, which are often the first point of contact, should be engaged more actively through simple reporting pathways, incentives, or recognition mechanisms. Strengthening the community and private sector will enable earlier identification and reporting of suspected events or potential public health threats.

Second, there is a need to strengthen the role of clinical surveillance focal points and specimen logistics. Notification delays were often linked to the absence of dedicated surveillance staff and challenges in sample collection and transport, and laboratory infrastructure. It is essential to ensure that all hospitals at all levels have trained focal points responsible for surveillance reporting and adequate laboratory infrastructure. In the absence of adequate laboratory infrastructure at subnational levels, policies to support timely specimen transport by improving logistics infrastructure, having guidelines which standardize urgent sample labeling and formalizing agreements with courier services must be created. These actions will help to improve the speed and quality of disease confirmation and notification.

Third, a strong multisectoral response capacity with clear governance and accountability must be developed and maintained. An effective and timely response requires coordination across sectors, particularly for One Health events. SOPs for activating multisectoral rapid response teams should be developed in the local language, disseminated, and enforced. Regular joint meetings, common communication platforms, and predefined roles and responsibilities are critical to sustain the achievement of the early response target. This is particularly important for vector-borne diseases such as dengue, which requires coordinated actions across multiple sectors, as the median (range) time to complete response actions was 51 (1–101) days. In parallel, strong governance structures and accountability mechanisms should be institutionalized. Clearly defined roles and responsibilities for each of the seven response actions across national and subnational levels, alongside transparent leadership and coordination processes, will enhance Cambodia’s ability to provide rapid and effective responses to public health events.

Fourth, there is a need to provide repeated training, supportive supervision, and technical support from the national level. Sustaining 7-1-7 readiness and motivation requires continuous capacity building and timely dissemination of technical updates. Regular mentoring from national trainers with short video-based modules and updated training materials in the local language can enhance preparedness and build confidence. Supervision visits from the national level can help monitor performance, provide on-the-job support, and reinforce best practices at the subnational level.

Fifth is the need to digitally integrate 7-1-7 indicators into the existing surveillance system. A key enabler for sustaining 7-1-7 performance is the integration of its metrics into Cambodia’s digital surveillance platform, CamEMS, which was very well highlighted in all qualitative interviews and discussions. Including standard date fields for detection, notification, and each of the seven response actions will allow for real-time monitoring. Dashboards showing event-specific and district-level performance can help identify delays and bottlenecks quickly at subnational levels.

The final and most important implication is to provide tailored interventions at the subnational level and secure adequate financial support. Study findings demonstrated clear variation in the achievement of 7-1-7 targets across events and provinces, with different levels exhibiting distinct performance patterns and operational needs. Some provinces consistently performed better than others, underscoring the importance of avoiding a one-size-fits-all approach. Subnational health systems should be supported through targeted improvement plans that reflect local epidemiology, response capacity, and past performance. Effective adoption and implementation also require secure and sustainable financing. Accessing funds and obtaining timely reimbursement were reported as frequent challenges in the implementation of the tool. Policies must therefore ensure access to flexible contingency funds for emergencies, along with streamlined reimbursement mechanisms. Such measures would enhance timeliness, reduce the financial burden on local teams, and maintain staff morale for efficient implementation of the 7-1-7 framework.

## Conclusion

Cambodia has successfully adopted the 7-1-7 framework at the national level, with subnational adoption in progress. The events reported in the public health sector demonstrated achievement in notification and early response targets, but detection timeliness remained a major challenge. Delays were mainly due to late health care-seeking and weak private sector reporting. Performance also varied across event types and provinces, pointing to the need for locally tailored solutions. To strengthen 7-1-7 implementation, Cambodia should focus on improving early detection through community and private sector engagement, ensuring trained staff and lab support at all levels, providing regular training and supervision, securing funding for rapid response, and integrating 7-1-7 indicators into the existing digital systems.

## Data Availability

The quantitative data supporting the findings of this study are derived from government health system sources in Cambodia and cannot be made freely available due to data ownership and confidentiality restrictions. However, de-identified qualitative and quantitative data can be provided to the journal for peer review and to readers upon reasonable request to the corresponding author ( sokly.moh@gmail.com). Assess requires approval from institution, and the authors can provide the datasets within 1–2 weeks of receiving the request. Zenodo: Adoption and use of the 7-1-7 metrics for effective detection, notification, and early response actions to public health events: A mixed-methods study in Cambodia, January 2023 to July 2025 All materials that can be made publicly available have been deposited as supplementary files in Zenodo and are openly available under the DOI: https://doi.org/10.5281/zenodo.18228118 ^30^. Data are available under the terms of the Creative Commons Attribution 4.0 International license (CC-BY 4.0). Following documents have been deposited (Zenodo) DOI: https://doi.org/10.5281/zenodo.18228118 ^30^ Supplementary file 1: Characteristics of Focus Group Discussions (FGDs) on Public Health Events, Cambodia, February–May 2025 Supplementary file 2: Characteristics of Key Informant Interviewees on Public Health Events, Cambodia, 2025 Suplimentary file 3-KII and fill-4: Questionnires for objective 1 and 2 Data are available under the terms of the Creative Commons Attribution 4.0 International license (CC-BY 4.0). Zenodo: COREQ and STROBE checklist for “Adoption and use of the 7-1-7 metrics for effective detection, notification, and early response actions to public health events: A mixed-methods study in Cambodia, January 2023 to July 2025” DOI: https://doi.org/10.5281/zenodo.18228118 ^30^. Data are available under the terms of the Creative Commons Attribution 4.0 International license (CC-BY 4.0).
