# Supplementary material for: Adoption and use of the 7-1-7 metrics for effective detection, notification, and early response actions to public health events: A mixed-methods study in Cambodia, January 2023 to July 2025
Source: Wellcome Open Res. 2026 Mar 12;10:673. Originally published 2025 Dec 12. [Version 3] doi: 10.12688/wellcomeopenres.25041.3 (PMC12988362; doi:10.12688/wellcomeopenres.25041.3)
Supplement: Peer review history

# Reviewer report: Reviewer response for version 3

**DOI:** 10.21956/wellcomeopenres.28771.r150562

**Published:** 2026-03-13

**Authors:** Robyn Alders

**Affiliations:** 1 Development Policy Centre, Crawford School of Public Policy, Australian National University, Canberra, ACT, Australia

Thanks to the authors for addressing my comments. The manuscript is, in my opinion, now suitable for indexing.

Is the work clearly and accurately presented and does it cite the current literature?

Partly

If applicable, is the statistical analysis and its interpretation appropriate?

Yes

Are all the source data underlying the results available to ensure full reproducibility?

Yes

Is the study design appropriate and is the work technically sound?

Partly

Are the conclusions drawn adequately supported by the results?

Partly

Are sufficient details of methods and analysis provided to allow replication by others?

Partly

Reviewer Expertise:

One Health, sustainable control of infectious diseases in small-scale livestock, household food and nutrition security

I confirm that I have read this submission and believe that I have an appropriate level of expertise to confirm that it is of an acceptable scientific standard.

# Reviewer report: Reviewer response for version 2

**DOI:** 10.21956/wellcomeopenres.28467.r145691

**Published:** 2026-02-16

**Authors:** Robyn Alders

**Affiliations:** 1 Development Policy Centre, Crawford School of Public Policy, Australian National University, Canberra, ACT, Australia

The authors are to be congratulated for conducting this research, it is very important to assess the utility of new metrics and targets.

 For a number of years, Cambodia has been burdened by a high prevalence of zoonotic diseases, such as high pathogenicity avian influenza (subtype H5N1) and rabies. The recognition of adopting a One Health approach is to be commended. 

 Aspects of the article that would benefit from attention include:

 * Participant genders were not recorded.  When dealing with rural households, small-scale poultry production is often under the management of women.  When gender disaggregated participant data is not included, it is difficult to assess the representativeness of the sample;

 * Timeliness of reporting was identified as a key factor. It would be ideal for the authors to discuss how the findings may have been enriched through the involvement of a One Health research team. While not the primary objective of this research, the identification of the importance of "a strong multisector response" could help to answer questions such as, why do small-scale poultry producers perceive high poultry mortality as normal? [The following questions are aspects you may wish to consider moving forward, and need not be addressed in this article. Would improved control of vaccine-preventable endemic animal diseases (such as fowl cholera and Newcastle disease) in small-scale poultry reduce the frequency of high poultry mortality events and improve relations between producers and veterinary services? When RRT reporting focuses on public health metrics, are they missing key drivers in community behaviour that contribute to a lack of reporting? Has the public health community adequately reflected on the impact of previous rounds of culling poultry infected with HPAI H5N1 without providing compensation to owners?]

 * In terms of the epidemiological analysis and laboratory diagnosis, is data also recorded for poultry samples that were negative for HPAI? The public health focus on zoonotic diseases is understandable; however, an interest in working with animal health colleagues to improve the overall health of animals would support a reduction in zoonotic disease prevalence and improved household food security;

 * Was data collected on the vaccination status of dogs (against rabies) and poultry (against fowl cholera and Newcastle disease)? If so, would it be possible seek correlations between reporting speed in relation to animal vaccination status?

 Once again, thank you for implementing and sharing the findings this research.

Is the work clearly and accurately presented and does it cite the current literature?

Partly

If applicable, is the statistical analysis and its interpretation appropriate?

Yes

Are all the source data underlying the results available to ensure full reproducibility?

Yes

Is the study design appropriate and is the work technically sound?

Partly

Are the conclusions drawn adequately supported by the results?

Partly

Are sufficient details of methods and analysis provided to allow replication by others?

Partly

Reviewer Expertise:

One Health, sustainable control of infectious diseases in small-scale livestock, household food and nutrition security

I confirm that I have read this submission and believe that I have an appropriate level of expertise to confirm that it is of an acceptable scientific standard, however I have significant reservations, as outlined above.

## References

[1] : Interventions to Reduce Risk for Pathogen Spillover and Early Disease Spread to Prevent Outbreaks, Epidemics, and Pandemics. *Emerging Infectious Diseases* .2023;29(3) : 10.3201/eid2903.221079 1-9 10.3201/eid2903.221079 PMC997369236823026

# Reviewer report: Reviewer response for version 2

**DOI:** 10.21956/wellcomeopenres.28467.r145125

**Published:** 2026-01-20

**Authors:** Dominic Delport

**Affiliations:** 1 Burnet Institute, Melbourne, Australia

I Approved. I have no further comments.

Is the work clearly and accurately presented and does it cite the current literature?

Yes

If applicable, is the statistical analysis and its interpretation appropriate?

Yes

Are all the source data underlying the results available to ensure full reproducibility?

Yes

Is the study design appropriate and is the work technically sound?

Yes

Are the conclusions drawn adequately supported by the results?

Yes

Are sufficient details of methods and analysis provided to allow replication by others?

Partly

Reviewer Expertise:

Infectious diseases epidemiology, epidemiological modelling, outbreak response modelling

I confirm that I have read this submission and believe that I have an appropriate level of expertise to confirm that it is of an acceptable scientific standard.

# Reviewer report: Reviewer response for version 1

**DOI:** 10.21956/wellcomeopenres.27607.r144014

**Published:** 2026-01-10

**Authors:** Dominic Delport

**Affiliations:** 1 Burnet Institute, Melbourne, Australia

I would like to thank the authors for putting together such a clear, insightful, and useful analysis. This mixed methods analysis of Cambodia's progress in adopting the 7-1-7 targets at different administrative levels, and identification of bottlenecks/enablers to the adoption process, provides clear evidence and steps for improvement. Cambodia's progress towards consistently meeting the 7-1-7 targets and the highlighted areas for improvement can be useful evidence to promote uptake of the targets and facilitate further progress in Cambodia.

 The authors have grounded this work well in the existing literature, and the findings and implications align well with other One Health and outbreak response timeliness analyses. In saying this, I have a few suggestions for additional references which the authors may find useful for their Background/Discussion sections, as they expand on and support many of the key ideas presented.

- 'One Health timeliness metrics to track and evaluate outbreak response reporting: A scoping review' by Fieldhouse et al. (2022)
- 'Using Timeliness Metrics to Track Progress and Identify Gaps in Disease Surveillance' by Crawley et al (2021)
- 'Finding Outbreaks Faster' by Smolinski et al (2017)
- 'How feasible or useful are timeliness metrics as a tool to optimise One Health outbreak responses?' by Fieldhouse et al (2024)

The study design and methods are appropriate and well-developed for this analysis. The only question I have is if there was a script or guide used during the Focus Group Discussions and Key Informant Interviews to ensure that consistent topics and questions where touched on in all sessions? I assume that Table 2 represents the primary questions/responses covered during the interviews? If so, could the script/questionnaire be provided in order to support the reproducibility of this kind of work?

 The Results and Discussion sections are clear and well structured, and the findings support the primary points of discussion and the conclusions drawn by the authors. 

 Congratulations and well done on this work! I just have a few more minor points to raise that I noticed while reading this paper, and if addressed I think they would serve to strengthen the piece and improve clarity/readability in a few areas.

- Background, paragraph 4 could use some supporting references for some of the specific statements.
- Background, paragraph 4 has a typo, I believe that “foodborne ill” should be “foodborne illnesses”?
- Methods, the sentence “The public health system is organized into three levels: National, Provincial, and Operational District (OD), Health Center level.” is awkward because of the third comma after “(OD)”. It reads to me like “Health Center level” is part of the list, which does not make sense? Does it mean that the public health system is organised into national health centers, provincial health centers, and OD health centers? Please clarify the sentence.
- Methods, Management of public health events in Cambodia, in paragraph 2 you refer to a “One Health RRT”, which is the first mention of One Health in the paper. It would likely benefit readers who may be unfamiliar with One Health as a concept to briefly discuss it in the Background section.
- Methods, Management of public health events in Cambodia, in paragraph 3 you mention that events are classified into risk levels which inform how they are handled. I would be interested to see a bit more discussion about this classification process, even at a high-level. What are the primary drivers for an event being high-risk vs low-risk, and does this risk assessment have potential effects on response timeliness?
- Results, Objective 1- Adoption of 7-1-7 metrics, in paragraph 1 you refer to “Government champion and coordination teams” but it is unclear to me what these are or how they impact 7-1-7 adoption at the national level relative to the subnational. In the Background you state that the CCDC Director is the “National 7-1-7 Champion” – is this the same as a “Government champion”? As these appear to be key concepts for the results, I think it would be useful to define them in the Methods section.

Is the work clearly and accurately presented and does it cite the current literature?

Yes

If applicable, is the statistical analysis and its interpretation appropriate?

Yes

Are all the source data underlying the results available to ensure full reproducibility?

Yes

Is the study design appropriate and is the work technically sound?

Yes

Are the conclusions drawn adequately supported by the results?

Yes

Are sufficient details of methods and analysis provided to allow replication by others?

Partly

Reviewer Expertise:

Infectious diseases epidemiology, epidemiological modelling, outbreak response modelling

I confirm that I have read this submission and believe that I have an appropriate level of expertise to confirm that it is of an acceptable scientific standard.
